# Reply to Landry et al. Findings from Diet Comparison Difficult to Interpret in the Absence of Adherence Assessment. Comment on “Tricò et al. Effects of Low-Carbohydrate versus Mediterranean Diets on Weight Loss, Glucose Metabolism, Insulin Kinetics and β-Cell Function in Morbidly Obese Individuals. *Nutrients* 2021, *13*, 1345”

**DOI:** 10.3390/nu13113695

**Published:** 2021-10-21

**Authors:** Domenico Tricò, Diego Moriconi, Monica Nannipieri

**Affiliations:** 1 Department of Surgical, Medical and Molecular Pathology and Critical Care Medicine, University of Pisa, 56126 Pisa, Italy; diego.moriconi@yahoo.com; 2 Department of Clinical and Experimental Medicine, University of Pisa, 56126 Pisa, Italy; monica.nannipieri@unipi.it

We thank Dr. Landry and colleagues [1] for their interest in our recent paper [2] and their comments.

Adherence to dietary prescriptions is of pivotal importance in studies comparing different nutritional interventions. In our randomized clinical trial, self-reported dietary compliance was assessed by trained dietitians at 2 and 4 weeks of diet using semiquantitative food frequency questionnaires, according to previously described methods [3,4]. Nutritional recommendations were carefully revised at 2 weeks, reducing the prescribed daily calorie intake in case of insufficient weight loss (≤2%) and reinforcing the instructions on the macronutrient composition of the diet if a ≥5% difference emerged between the prescribed and actual macronutrient intake. Due to the strict monitoring and individualized counseling, the estimated adherence to the energy and macronutrient composition of the diet was >85% in all participants at the end of the intervention. The fact that the actual weight loss was close to the predicted in most participants, in the absence of substantial changes in physical activity, further confirms the good adherence to dietary energy prescriptions in both study arms, being in fact the achieved weight loss an objective measure of the daily energy deficit. 

As already pointed out and discussed [2], a low-carbohydrate/high-protein diet has generally demonstrated greater effectiveness in reducing body weight, particularly in short term studies. It is not surprising therefore—and perhaps a further clue of the good adherence to the diet—that volunteers randomized to the low-carbohydrate diet lost more weight than those following a balanced Mediterranean diet. Whether the higher efficacy of the low-carbohydrate/high-protein diet is driven by the peculiar properties of proteins [5,6,7,8,9] or could be partly attributed to greater engagement and adherence of participants, as suggested by the Authors [1], cannot be established from our data. However, a different engagement between the two study groups seems unlikely given that all participants were extremely motivated to achieve a minimum weight loss of 5%, regardless of the type of diet, as strictly required to qualify for future metabolic surgery. 

Lastly, participants’ satisfaction with their assigned diet was not formally assessed and quantified in our study. However, none withdrew the diet or complained about its composition during the 4-week intervention, suggesting the feasibility of these dietary prescriptions, at least in the short term. 

In conclusion, our study emphasizes important differences in the effectiveness of two calorie-restricted eating patterns on weight loss and glucose homeostatic mechanisms in morbidly obese individuals. A great effort has been made to maximize participants’ engagement to nutritional interventions and reduce heterogeneity, with the final aim to minimize the influence of dietary adherence on study findings.

## Data Availability

Not applicable.

## References

[1] Landry M.J., Crimarco A., Gardner C.D. (2021). Findings from diet comparison difficult to interpret in the absence of adherence assessment. Comment on Tricò et al. Effects of low-carbohydrate versus mediterranean diets on weight loss, glucose metabolism, insulin kinetics and β-cell function in morbidly obese individuals. *Nutrients* 2021, *13*, 1345. Nutrients.

[2] Tricò D., Moriconi D., Berta R., Baldi S., Quinones-Galvan A., Guiducci L., Taddei S., Mari A., Nannipieri M. (2021). Effects of low-carbohydrate versus mediterranean diets on weight loss, glucose metabolism, insulin kinetics and β-cell function in morbidly obese individuals. Nutrients.

[3] Tricò D., Trifiro S., Mengozzi A., Morgantini C., Baldi S., Mari A., Natali A. (2018). Reducing cholesterol and fat intake improves glucose tolerance by enhancing beta cell function in nondiabetic subjects. J. Clin. Endocrinol. Metab..

[4] Morgantini C., Trifiro S., Trico D., Meriwether D., Baldi S., Mengozzi A., Reddy S.T., Natali A. (2018). A short-term increase in dietary cholesterol and fat intake affects high-density lipoprotein composition in healthy subjects. Nutr. Metab. Cardiovasc. Dis..

[5] Eisenstein J., Roberts S.B., Dallal G., Saltzman E. (2002). High-protein weight-loss diets: Are they safe and do they work? A review of the experimental and epidemiologic data. Nutr. Rev..

[6] Bray G.A., Smith S.R., de Jonge L., Xie H., Rood J., Martin C.K., Most M., Brock C., Mancuso S., Redman L.M. (2012). Effect of dietary protein content on weight gain, energy expenditure, and body composition during overeating: A randomized controlled trial. JAMA.

[7] Tricò D., Biancalana E., Solini A. (2021). Protein and amino acids in nonalcoholic fatty liver disease. Curr. Opin. Clin. Nutr. Metab. Care.

[8] Stentz F.B., Mikhael A., Kineish O., Christman J., Sands C. (2020). High protein diet leads to prediabetes remission and positive changes in incretins and cardiovascular risk factors. Nutr. Metab. Cardiovasc. Dis..

[9] Nesti L., Mengozzi A., Tricò D. (2019). Impact of Nutrient Type and Sequence on Glucose Tolerance: Physiological Insights and Therapeutic Implications. Front. Endocrinol..

